# The motility regulator *flhDC* drives intracellular accumulation and tumor colonization of *Salmonella*

**DOI:** 10.1186/s40425-018-0490-z

**Published:** 2019-02-12

**Authors:** Vishnu Raman, Nele Van Dessel, Owen M. O’Connor, Neil S. Forbes

**Affiliations:** Department of Chemical Engineering, University of Massachusetts, 159 Goessmann Laboratory, 686 North Pleasant St, Amherst, MA 01003 USA

**Keywords:** *Salmonella*, Bacterial cancer therapy, Cancer therapy, Intracellular invasion, Intracellular cancer therapy

## Abstract

**Background:**

*Salmonella* have potential as anticancer therapeutic because of their innate tumor specificity. In clinical studies, this specificity has been hampered by heterogeneous responses. Understanding the mechanisms that control tumor colonization would enable the design of more robust therapeutic strains. Two mechanisms that could affect tumor colonization are intracellular accumulation and intratumoral motility. Both of these mechanisms have elements that are controlled by the master motility regulator *flhDC*. We hypothesized that 1*) overexpressing flhDC in Salmonella increases intracellular bacterial accumulation in tumor cell masses,* and *2) intracellular accumulation of Salmonella drives tumor colonization in vitro*.

**Methods:**

To test these hypotheses, we transformed *Salmonella* with genetic circuits that induce *flhDC* and express green fluorescent protein after intracellular invasion. The genetically modified *Salmonella* was perfused into an *in vitro* tumor-on-a-chip device. Time-lapse fluorescence microscopy was used to quantify intracellular and colonization dynamics within tumor masses. A mathematical model was used to determine how these mechanisms are related to each other.

**Results:**

Overexpression of *flhDC* increased intracellular accumulation and tumor colonization 2.5 and 5 times more than control *Salmonella*, respectively (P < 0.05). Non-motile *Salmonella* accumulated in cancer cells 26 times less than controls (P < 0.001). Minimally invasive, *ΔsipB*, *Salmonella* colonized tumor masses 2.5 times less than controls (P < 0.05). When *flhDC* was selectively induced after penetration into tumor masses, *Salmonella* both accumulated intracellularly and colonized tumor masses 2 times more than controls (P < 0.05). Mathematical modeling of tumor colonization dynamics demonstrated that intracellular accumulation increased retention of *Salmonella* in tumors by effectively causing the bacteria to bind to cancer cells and preventing leakage out of the tumors. These results demonstrated that increasing intracellular bacterial density increased overall tumor colonization and that *flhDC* could be used to control both.

**Conclusions:**

This study demonstrates a mechanistic link between motility, intracellular accumulation and tumor colonization. Based on our results, we envision that therapeutic strains of *Salmonella* could use inducible *flhDC* to drive tumor colonization. More intratumoral bacteria would enable delivery of higher therapeutic payloads into tumors and would improve treatment efficacy.

**Electronic supplementary material:**

The online version of this article (10.1186/s40425-018-0490-z) contains supplementary material, which is available to authorized users.

## Introduction

Effective tumor colonization is essential for bacterial anti-cancer therapy. With poor colonization, insufficient treatment is delivered and the tumor response is hampered. For bacterial therapy, the tumor density is controlled more by the rate of colonization than the administered dose [1]. However, the mechanisms that control colonization are poorly understood. It has been well established that after intravenous injection into mice, *Salmonella* colonizes tumor tissue at ratios greater than 10,000:1 compared to other organs in the body [2]. It is this tumor specificity that makes *Salmonella*-based therapy particularly attractive as a targeted delivery agent [3]. Unfortunately, clinical trials showed that tumor colonization in humans was not sufficient to induce a lasting response [4]. Therefore, understanding and controlling the mechanisms that drive bacterial tumor colonization could greatly improve bacterial tumor therapy.

Two mechanisms that could affect tumor colonization are intratumoral motility and intracellular accumulation. We have previously shown that bacterial motility plays a critical role in the accumulation of *Salmonella* in tumors [5–7]. Upregulating motility by swim-plate selection increases distal tumor colonization of the bacteria [6, 8] and altering chemotactic sensing increases bacterial penetration into tumor masses [7–9]. *Salmonella* motility is controlled by the master regulator *flhDC* [10–12]. The *flhDC* protein complex regulates expression of the functional flagellar components [13]. This regulator is one of the most tightly regulated transcription factors within bacteria [14–19]. Flagella-dependent motility is downregulated under nutrient deprivation in *Salmonella*, which helps *Salmonella* survive intracellularly where there is limited availability of nutrients [20].

Intracellular invasion and growth are important mechanisms that could also affect *Salmonella* colonization of tumors. *Salmonella* have two type three secretion systems, T3SS1 and T3SS2, that promote invasion, survival, and growth inside epithelial cells [21]. Other *Salmonella* invasion systems include the *Rck* system, which invades cells by binding to epidermal growth factor receptor [22]. In the gut, *Salmonella* use these systems to invade and grow inside intestinal cells [23]. Disabling T3SS2 limits the ability of *Salmonella* to inhibit tumor growth [24]. When T3SS2 genes are deleted by transposon insertion, bacterial accumulation in the spleen is reduced [25]. After serial passaging in mice, *Salmonella* with increased intracellular invasion had enhanced persistence [26]. We have seen similar effects in tumor cell masses *in vitro*. Compared to K-12 *E. Coli* that is T3SS deficient, *Salmonella* had considerably greater colonization [5].

The two *Salmonella* secretions systems have distinct functions. T3SS1 initiates invasion into epithelial cells and T3SS2 enables intracellular growth and survival [21]. Both systems are composed of a needle apparatus that spans the inner and outer membranes, and the peptidoglycan layer [27]. Effector proteins are injected into the mammalian cells through the T3SS1 [27, 28]. Secretion of T3SS1 effectors into mammalian cell cytoplasm is required for T3SS dependent intracellular invasion of *Salmonella* [29]. Once injected, these effectors cause a rearrangement of the mammalian actin cytoskeleton and endocytosis of *Salmonella* [30, 31]. One essential effector protein is *sipB*. When knocked out, *Salmonella* cannot invade using T3SS1 [32]. When *Salmonella* have internalized, the bacteria modify the endocytic vacuole by secreting T3SS2 effectors [33–35]. These modifications confer protection to the bacteria and enable intracellular growth and survival [36, 37]. The T3SS-dependent intracellular invasion and survival of *Salmonella* confers protection against extracellular clearance mechanisms, like compliment and attack by macrophages and neutrophils [23, 38]. A non-functional T3SS2 apparatus impairs *in vivo* colonization and anti-tumor efficacy of *Salmonella* [24, 25], indicating the importance of intracellular growth for survival of bacteria *in vivo*.

Flagella-dependent motility and intracellular invasion are not regulated independently. Rather, both of these systems are intertwined and there is a complex feedback between them [39, 40]. Increasing bacterial motility also increases intracellular invasion [41]. The *flhDC* transcriptional complex controls elements of both motility and cellular invasion. In addition to controlling expression of motility genes, it directly controls the expression of the dual regulatory element, *fliZ*. FliZ controls both flagellar hook assembly and upregulates the transcription factor *hilD* [39–41]. *HilD* expression directly upregulates T3SS1 expression and intracellular invasion [39, 40]. The systems are further connected because flagella can act as physical cell surface sensors to determine the optimal extracellular location to initiate invasion [42]. These systems are connected in part because the T3SS evolved from the flagellar type three secretion system (fT3SS), which is used to assemble functional flagella [43, 44]. The co-regulation of motility and intracellular invasion further supports the idea that both of these phenomena are important for bacterial tumor colonization.

In addition to affecting intracellular invasion, flagella-dependent motility also affects the intracellular lifestyle of *Salmonella*. Immediately after invasion the majority of *Salmonella* reside in intracellular vacuoles. A small but significant fraction of the intracellular bacteria escape from the vacuoles into the cytosol [45–47]. Some cytosolic bacteria are degraded by host ubiquitination machinery [48–52]. Those that escape degradation replicate rapidly and are extruded from the cell [45]. The T3SS1 system and functional flagella play important roles in the escape from the vacuole and the hyper-replication [45–47]. After extrusion, the bacteria are primed for reinvasion because of the expression of flagella and SPI-I invasion genes [45, 46].

The goal of this study was to measure the effect of intracellular accumulation on bacterial tumor colonization and quantify the interplay between intracellular accumulation and motility. The interaction of these mechanisms has not been previously studied in relation to using bacteria for cancer therapy. We hypothesized that 1) *overexpressing flhDC in Salmonella increases intracellular accumulation in tumor cell masses*, and 2) *intracellular accumulation of Salmonella drives tumor colonization in vitro*. To test these hypotheses, *Salmonella* were transformed with genetic circuits that induce *flhDC and* express green fluorescent protein (*GFP*) after cell invasion. Genetically modified *Salmonella* were perfused into a microfluidic tumor-on-a-chip device to assess colonization and invasion using time-lapse fluorescence microscopy. The potential to use *flhDC* as a bispecific switch to increase tumor colonization was determined by inducing expression after initial penetration. A mathematical model was used to investigate why intracellular invasion and growth improved tumor colonization of *Salmonella*. Controlling *Salmonella* invasion into cells will increase overall tumor colonization and has the potential to make these therapeutic bacteria more effective in the clinic.

## Materials and Methods

### Bacterial Strains and Plasmid Construction

Eight strains of *Salmonella Enterica* serovar Typhimurium were used throughout the experiments (Table 1). The control strain (Sal) was based on an attenuated therapeutic strain of *Salmonella* (VNP20009) that has three deletions, *ΔmsbB, ΔpurI, and Δxyl*, that eliminate most toxicities *in vivo*. The background strain was transformed with a plasmid containing two gene circuits, *P*_*lac*_*/DsRed and P*_*SSEJ*_*/GFP*, that constitutively express *DsRed* and express *GFP* after intracellular invasion (Table 1; Additional file 1: Figure S1-A). The constitutive lac *DsRed* gene circuit was created by adding the wild-type lac promoter and a ribosomal binding site (AAGGAG) to the 5’ end of the forward *DsRed* primer. The *SSEJ* promoter was copied by PCR from VNP20009 genomic DNA using the following primers: forward-ACATGTCACATAAAACACTAGCACTTTAGC and reverse- TCTAGACCTCCTTACTTTATTAAACACGCT. The second strain, F-Sal, was transformed with a plasmid that contains a third gene circuit that enables induction of *flhDC* with arabinose (Table 1; Additional file 1: Figure S1-B). PCR was used to amplify the *flhDC* genes from *Salmonella* genomic DNA using the following primers: forward-AAAAAACCATGGGTTAATAAAAGGAGGAATATATATGCATACATCCGAGTTGCTAAAACA and reverse- AAAAAACTCGAGAAAAATTAAACAGCCTGTTCGATCTGTTCAT. The PCR product and PBAD-his-myc plasmid (*Invitrogen*, Carlsbad, CA) were digested with NcoI and XhoI and ligated with T4 DNA ligase. The *flhDC* expression cassette, which includes the AraC regulator and PBAD controlled *flhDC*, was amplified with PCR and combined with a plasmid containing SSEJ-*GFP* and Lac-*DsRed* using Gibson Assembly. Both S-Sal, which has a *sipB* deletion, and the *ΔflgE* strain were generated using lambda red recombination [53]. When the flagellar hook (*flgE*) is deleted, *Salmonella* are unable to produce functional flagella and are non-motile [54]. The S-Sal strain (strain three) was transformed with the plasmid containing *P*_*lac*_*/DsRed and P*_*SSEJ*_*/GFP* (Table 1; Additional file 1: Figure S1-A). The fourth strain, FS-Sal, was transformed with a plasmid that contains inducible *flhDC* (*P*_*BAD*_*/flhDC*), constitutive *DsRed* expression (*P*_*lac*_*/DsRed*) and intracellular *GFP* expression (*P*_*SSEJ*_*/GFP*) in a Δ*sipB* background (Table 1; Additional file 1: Figure S1-B). A second control *Salmonella* strain (strain five) was transformed with a plasmid containing *P*_*lac*_*/GFP* to constitutively express *GFP* (Table 1; Additional file 1: Figure S1-C). The constitutive lac *GFP* gene circuit was created similarly to the lac *DsRed* circuit, by adding the wild-type lac promoter and a ribosomal binding site (AAGGAG) to the 5’ end of the forward *GFP* primer. The sixth strain, *Salmonella+pflhDC*, expresses *GFP* constitutively and *flhDC* upon induction with arabinose (Table 1; Additional file 1: Figure S1-D). The seventh strain, *ΔflgE*, is non-motile and expresses *GFP* constitutively (Table 1; Additional file 1: Figure S1-C). The eighth strain, *ΔflgE*+*pflhDC*, expresses *GFP* constitutively and *flhDC* upon induction with arabinose (Table 1; Additional file 1: Figure S1-D). All cloning was performed with DH5α *E. Coli* (*New England Biolabs*, Ipswich, MA) and all plasmids contained a ColE1 origin and either chloramphenicol or ampicillin resistance (Additional file 1: Figure S1). *Salmonella* were transformed by electroporation. All cloning reagents, buffer reagents, and primers were from *New England Biolabs*, *Fisher Scientific* (Hampton, NH), and *Invitrogen*, (Carlsbad, CA), respectively, unless otherwise noted.

**Table 1:** *Salmonella* strains and plasmids

Strain	Background	Genetic circuits	Description
1. Sal	*ΔmsbB*, *ΔpurI*, *Δxyl* (VNP20009)	*P*_*lac*_ */DsRed P*_*SSEJ*_ */GFP*	Constitutive *DsRed* Intracellularly inducible *GFP* Additional file 1: Figure S1-A
2. F-Sal	Sal	*P*_*lac*_ */DsRed P*_*SSEJ*_ */GFP P*_*BAD*_ */flhDC*	Arabinose Inducible *flhDC* Constitutive *DsRed* Intracellularly inducible *GFP* Additional file 1: Figure S1-B
3. S-Sal	Δ*sipB* Sal	*P*_*lac*_ */DsRed P*_*SSEJ*_ */GFP*	Minimally Intracellularly Invasive Constitutive *DsRed* Intracellularly inducible *GFP* Additional file 1: Figure S1-A
4. FS-Sal	Δ*sipB* Sal	*P*_*lac*_ */DsRed P*_*SSEJ*_ */GFP P*_*BAD*_ */flhDC*	Arabinose Inducible *flhDC* Minimally Intracellularly Invasive Constitutive *DsRed* Intracellularly inducible *GFP* Additional file 1: Figure S1-B
*5. Salmonella* *(control)*	*ΔmsbB*, *ΔpurI*, *Δxyl*	*P*_*lac*_ */GFP*	Constitutive GFP Additional file 1: Figure S1-C
*6. Salmonella + pflhDC*	*ΔmsbB*, *ΔpurI*, *Δxyl*	*P*_*lac*_ */GFP* *P*_*BAD*_ */flhDC*	Arabinose inducible *flhDC* Constitutive GFP Additional file 1: Figure S1-D
*7. ΔflgE*	*ΔmsbB*, *ΔpurI*, *Δxyl ΔflgE*	*P*_*lac*_ */GFP*	Non-motile Constitutive GFP Additional file 1: Figure S1-C
*8. ΔflgE+pflhDC*	*ΔmsbB*, *ΔpurI*, *Δxyl ΔflgE*	*P*_*lac*_ */GFP* *P*_*BAD*_ */flhDC*	Arabinose inducible *flhDC* Non-motile Constitutive GFP Additional file 1: Figure S1-D

### Cell Culture

MCF7 breast carcinoma cells and LS174T colorectal carcinoma cells (*ATCC*, Manassas, VA) were maintained in DMEM (Dulbecco's Modified Eagle Medium; *Sigma Aldrich*, St. Louis, MO) with 1 g/L glucose, 3.7 g/L sodium bicarbonate (pH 7.4) and 10% FBS using standard cell culture techniques. Between passages of LS174T cells, single cell suspensions were transferred to PMMA coated cell culture flasks (2 g/L PMMA in 100% ethanol, dried before use) in order to produce spheroids.

### Fabrication and Operation of Microfluidic Devices

Photolithography was used to make silicon wafer masters as previously described [55]. Two silicon wafers were made: One silicon wafer was used to make the pneumatic valve layer (layer 1). The other wafer was to make the media perfusion layer (layer 2). The fabrication of multi-layer tumor-on-a-chip devices was based on a previous method [56]. The microfluidic device was fabricated in two parts. Layer 1 was created by mixing 9 parts of Sylgard 184 PDMS (*Ellsworth Adhesives*, Wilmington, MA) with 1 part of curing agent and poured onto the pneumatic valve layer silicon master wafer. Layer 2 was created by mixing 15 parts of PDMS with 1 part (weight by mass) of curing agent and spun coat onto the media perfusion silicon wafer to a height of 200 μm. Both layers of PDMS were cured at 65 °C for 1.5 h and layer 1 was aligned on top of layer 2. Both layers were cured together at 95 °C for 1 h. Holes were punched in the PDMS layers to receive fluidic and control tubing. The PDMS layers were bonded to a glass slide by plasma treatment (Harrick Plasma Cleaner). The valves were pneumatically actuated before bonding to prevent the valve from sealing. Devices were taped to a microscope stage adaptor and inlet and outlet tubes were inserted. A 10% bleach solution was perfused at 3 μl/min throughout the device for 2 h followed by 70% ethanol for 1 h. The device was prepared for spheroid loading by perfusing for 1 h with DMEM with 1 g/L glucose, 20 mM HEPES (pH 7.4), 10% FBS and 33 μg/ml chloramphenicol (henceforth referred to as DMEM-HEPES-chlor). For all experiments, ~300 μm diameter LS174T spheroids were positioned into a microfluidic device and equilibrated in DMEM-HEPES-chlor for 6 h at a flow rate of 3 μl/min. Some spheroids were damaged in the insertion process and these cell masses were not included in the image analysis.

### Quantifying Intracellular Invasion and Colonization of *Salmonella* in a Tumor-on-a-chip

Four experiments were performed with tumor-on-a-chip device to quantify colonization and intracellular accumulation for (1) induced F-Sal compared to Sal, (2) FS-Sal compared to S-Sal, (3) S-Sal compared to Sal, and (4) for intratumoral induction of F-Sal compared to Sal. *Salmonella* strains were grown in LB with chloramphenicol (33 μg/ml) to a density of approximately 250 million CFU/ml. Bacteria were resuspended in DMEM-HEPES-chlor at a density of 10 million CFU/ml. The bacterial suspension was perfused into the tumor-on-a-chip device for 1h at a flowrate of 3 μl/min followed by bacteria-free DMEM-HEPES-chlor at the same flowrate for 48 h. In experiments one and two, the F-Sal and FS-Sal conditions contained 0.4% arabinose to induce *flhDC*. Flowing bacteria-fee medium prevents over growth in the flow channel and mimics clearance *in vivo*. For experiment four, the procedure was the same (bacterial perfusion for 1 h, followed by perfusion with bacteria-free medium), except that after 11 h, medium containing 0.4% arabinose was perfused into the device to induce *flhDC* intratumorally.

Transmitted and fluorescent images (480/525 excitation/emission for *GFP*mut3 and 525/590 for *DsRed*) of tumor masses were acquired every hour with an Olympus IX71 or a Zeiss Axio Observer Z.1 microscope. Time lapse microscopy images of each tumor mass were cropped using the rectangular cropping tool in ImageJ and were analyzed in Matlab. Each image was background subtracted. Fluorescent intensities of ten spatially equal sections of each tumor mass were averaged to quantify bacterial profiles for each time point. Overall bacterial density as a function of time was determined by averaging fluorescent intensities for entire tumor masses per time point. Red fluorescence was used to calculate total bacterial colonization and green fluorescence was used to calculate intracellular bacterial density. Each experiment was normalized by dividing every calculated average fluorescence intensity by the highest fluorescent intensity observed, which occurred during the last time point.

### Quantifying Aqueous Motility of *Salmonella*

Aqueous motility was determined by growing *flhDC* inducible *Salmonella* in 0.4% arabinose. Twenty microliters of 400 million CFU/ml of either *flhDC* induced or control *Salmonella* was placed between a coverslip and a glass slide. Transmitted light microscopy images were taken every 0.68 seconds for approximately 30 seconds. The automated particle tracking plugin in ImageJ, Trackmate, was used to analyze bacterial swimming velocity. Aqueous velocity histograms were generated by binning the fraction of total bacteria into three velocity categories: 0-15 μm/s, 15-30 μm/s and >30 μm/s. Motility assays were performed in triplicate.

### Quantifying Intracellular Invasion and Growth inside MCF7 Cells in Monolayer

Intracellular invasion of *Salmonella* was quantified by growing in LB and adding to monolayer cultures of MCF7 cancer cells. Four strains were used to quantify the dependence on *flhDC* expression and flagella formation: control *Salmonella*, *Salmonella+pflhDC*, *ΔflgE*, *ΔflgE+pflhDC*. Two strains were used to show the intracellular specificity of the *P*_*SSEJ*_ promoter and the dependence on T3SS: Sal and S-Sal, using a modified gentamycin protection assay. Each strain was grown in LB to a density of 5 × 10^8^ CFU/ml and added to 6-well plates of MCF7 cells at a density of 5 × 10^6^ CFU/ml. After two hours of incubation, each well was washed ten times with one milliliter of phosphate buffered saline. DMEM with 20 mM HEPES and 40 μg/ml gentamycin was added to each well to remove residual extracellular bacteria. For two hours following the addition of gentamycin, the cultures were observed microscopically to assess the effectiveness of the PBS washes to remove extracellular bacteria. The few remaining extracellular bacteria were observed over this period to ensure that they were eliminated by the gentamycin treatment. After two hours, intracellular *Salmonella* were imaged over time at 10X magnification with fluorescence microscopy. After 18 hours, bacterial invasion was quantified by randomly identifying 20 cells in each culture and counting the fraction of cells that contained intracellular *Salmonella,* as indicated by *GFP* fluorescence.

A similar invasion protocol was used to calculate the intracellular growth rate of *Salmonella*. Both control *Salmonella* and *Salmonella+pflhDC* constitutively expressed *GFP* (Table 1). Time lapse fluorescence microscopy was used to quantify the fluorescence from *P*_*lac*_*/GFP Salmonella* inside MCF7 cells over time. Salmonella density was determined by multiplying the average intensity by the area of all intracellular bacteria within a cell, as a function of time. It was assumed that the amount of *GFP* produced per bacterium was constant over time. Only MCF7 cells containing bacteria and that did not divide for a six hour interval were used. Intracellular growth rate was calculated by fitting an exponential growth function to the intracellular bacterial density.

### Mathematical Modeling

A mathematical model was created to interpret the spatiotemporal dynamics of bacterial dispersion, growth and invasion in tumor masses. This model was based on a previous model of bacterial growth in tumor tissue [57].

1 $$ \frac{{\partial c}_{ex}}{\partial t}=D\frac{\partial^2{c}_{ex}}{{\partial x}^2}+\frac{\partial }{\partial x}\left({k}_{aff}\frac{d{c}_{chem}}{dx}{c}_{ex}\right)+{\mu}_g{c}_{ex}-{\mu}_{inv}{c}_{ex}\theta $$

2 $$ \frac{{\partial c}_{in}}{\partial t}={\mu}_{g, in}{c}_{in}+{\mu}_{in v}{c}_{ex}\theta $$

3 $$ \operatorname{}{c}_{ex, in}{\left|{}_{t=0}=0\kern0.50em ,\operatorname{}\frac{{\partial c}_{ex}}{\partial t}\right|}_{x=0}=\frac{F_0}{V}\left({c}_{ex,0}-{c}_{ex}\right)+\frac{A}{V}D\operatorname{}\frac{{\partial c}_{ex}}{\partial x}{\left|{}_{x=0},\operatorname{}\frac{d{c}_{ex}}{\partial x}\right|}_{x=1}=0 $$

The coupled PDE model incorporated a balance on extracellular (eq. 1) and intracellular (eq. 2) bacteria. The balance for extracellular bacteria includes the effects of dispersion [$D\frac{\partial^2{c}_{ex}}{{\partial x}^2}$], chemotaxis [$\frac{\partial }{\partial x}\left({k}_{aff}\frac{d{c}_{chem}}{dx}{c}_{ex}\right)$], growth [*μ*_*g*_*c*_*ex*_], and invasion [*μ*_*inv*_*c*_*ex*_*θ*]. The intracellular balance includes the effects of intracellular growth [*μ*_*g*, *in*_*c*_*in*_] and invasion [*μ*_*inv*_*c*_*ex*_*θ*]. The initial and boundary conditions (eq. 3) state that (1) there were no intracellular or extracellular bacteria initially within the tumor mass; (2) the flux into or out of the tumor mass was equal to the flux in the supply channel; and (3) there was no flux at the distal (x = 1) boundary. The supply of extracellular bacteria (*C*_*ex,0*_) is a stepwise function that was set to match experimental conditions: 10^7^ CFU/ml of bacteria were administered for 2 h, followed by perfusion of bacteria-free media for the remaining time.

The variables in the model are as follows: *C*_*ex*_ and *C*_*in*_ are the normalized extracellular and intracellular densities (a value of one corresponds to 1x10^10^ CFU/ml), *D* is the dispersion coefficient, *μ*_*g*_ and *μ*_*g,in*_ are the extracellular and intracellular growth rates, *μ*_*inv*_ is the intracellular invasion rate, *θ* is the fraction of viable tumor cells, *K*_*aff*_ is the chemotactic affinity to chemokines in the tumor mass, *C*_*chem*_ is the normalized chemokine concentration, *C*_*ex,0*_ is the normalized density of bacteria that was perfused into the microfluidic device as a function of time (1x10^7^ CFU/ml for t ≤ 2 h and 0 for t > 2 h), *F*_*0*_ is the media flow rate in the perfusion channel, *V* is the volume of the section of the perfusion channel in front of the tumor chamber, and *A* is the cross-sectional area of the tumor chamber. All intracellular and total bacterial fluorescence values were normalized to the highest cross sectional fluorescence intensity that occurred during the experiment.

Equations were discretized in space and solved in *Matlab* (*The MathWorks, Inc.*, Natick, MA) using a finite difference method. The spatially discretized coupled ordinary differential equations were solved with the built-in ode15s function in *Matlab* for all spatial (discretized in ten points in space) and temporal points between 0 and 40 hours in 1 hour intervals. The fraction of viable cancer cells within the tumor mass (*θ*) was calculated based on previous data [9]. The extracellular growth rate was calculated based on the growth rate in liquid culture.

Two datasets (F-Sal vs. Sal and S-Sal vs. Sal) were used for modelling and normalized to one another to match control (Sal) conditions. The bacterial dispersion coefficient was calculated by fitting the model (eq. 1-3) to the tumor-on-a-chip experimental data of *GFP* for all spatial and temporal points up to 40 hours. The *fminsearch* function in *Matlab* was used to minimize the sum of least squares error between the experimental data and model by adjusting (and calculating) the rates of intracellular invasion and dispersion for both Sal datasets. The intracellular invasion rate of S-Sal was calculated by fixing the dispersion coefficient to be the same as Sal. The dispersion coefficient and intracellular invasion rate of F-Sal were calculated by bounding the dispersion coefficient such that it could not be lower than that of Sal. The intracellular accumulation rate was determined by quantifying the total change in intracellular density between 47 and 48 h.

### Data and Statistical Analysis

Image and statistical analysis was performed in *Matlab* software. Unpaired t-tests with unequal variance were used to determine statistical significance with a level of P < 0.05.

## Results

### Induction of *flhDC* increases tumor colonization of *Salmonella*

**Fig. 1:**
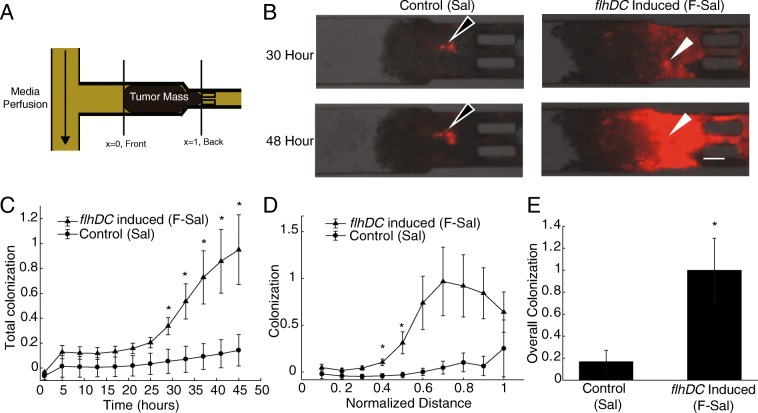
Inducing *Salmonella* with *flhDC* increase bacterial tumor colonization and dispersion. **a)** The microfluidic device contained a media perfusion channel and a chamber that holds tumor cell masses. The perfusion channel mimics tumor vasculature. Masses are formed as spheroids and inserted through tubing and control valves. Prior to insertion, spheroids are approximately 300 μm in diameter. **b)** Colonization of control Sal (*black arrows*) and *flhDC*-induced F-Sal (white arrows) was measured by with red fluorescence (*red*). Tumor cell masses are shown in the transmitted images under the fluorescence images. Images were background subtracted and shown with the maximum red intensity at the greatest observed value. Scale bar is 100 um. **c)** *Salmonella* with induced *flhDC* (F-Sal) colonized tumors significantly more than *Salmonella* (Sal) from 29 to 45 hours after bacterial administration (*, *P*<0.05, *n* = 3 for Sal and *n* = 5 for F-Sal). **d)** F-Sal colonized proximal (x≤0.5) tissue more than control *Salmonella* (Sal; *, *P*<0.05). The density was ten-fold greater for F-Sal in distal tumor tissue. **e)** At 48 hours after administration, F-Sal colonized tumors five-fold more control Sal (*, *P*<0.05).

Overexpressing *flhDC* in *Salmonella* increased intratumoral dispersion and colonization (Fig. 1). When administered to a tumor-on-a-chip device (Fig. 1A), F-Sal (induced *flhDC*) colonized tumor masses more than Sal (control) *Salmonella* (Fig. 1B). Both strains contained *P*_*lac*_*/DsRed* and expressed *DsRed* constitutively. In these images, red fluorescence indicates overall bacterial density. At 30 h, the size of the colony formed by F-Sal (*white arrows*) was considerably larger than the one formed by Sal (*black arrows*, Fig. 1B). The area of both colonies increased in size from 30 to 48 h after bacterial administration. Both colonies were located deep into the tissue, away from the perfusion channel (see Fig. 1A), indicating that both strains actively penetrated the tumor masses as we have described previously [5, 6]. Across multiple cell masses (*n* = 3 for Sal and *n* = 5 for F-Sal), the average density of F-Sal was significantly greater than Sal within entire tumor masses between 29 and 45 hours of bacterial colonization (P < 0.05; Fig. 1C). After 48 hours of bacterial colonization, F-Sal colonized both proximal (x ≤ 0.5) and distal (x = 0.9) tumor tissue more than Sal (P < 0.05; Fig. 1D). The density of F-Sal was greater than Sal throughout the middle of tumor masses (0.6 ≤ x 0.8), but was not significant (0.05 < P < 0.08) because of heterogeneous localization of colonies between cell masses (Fig. 1D). Overall, F-Sal colonized tumor tissue five-fold more than Sal (P < 0.05, Fig. 1E).

### Overexpression of *flhDC* increases intracellular accumulation of *Salmonella*

**Fig. 2:**
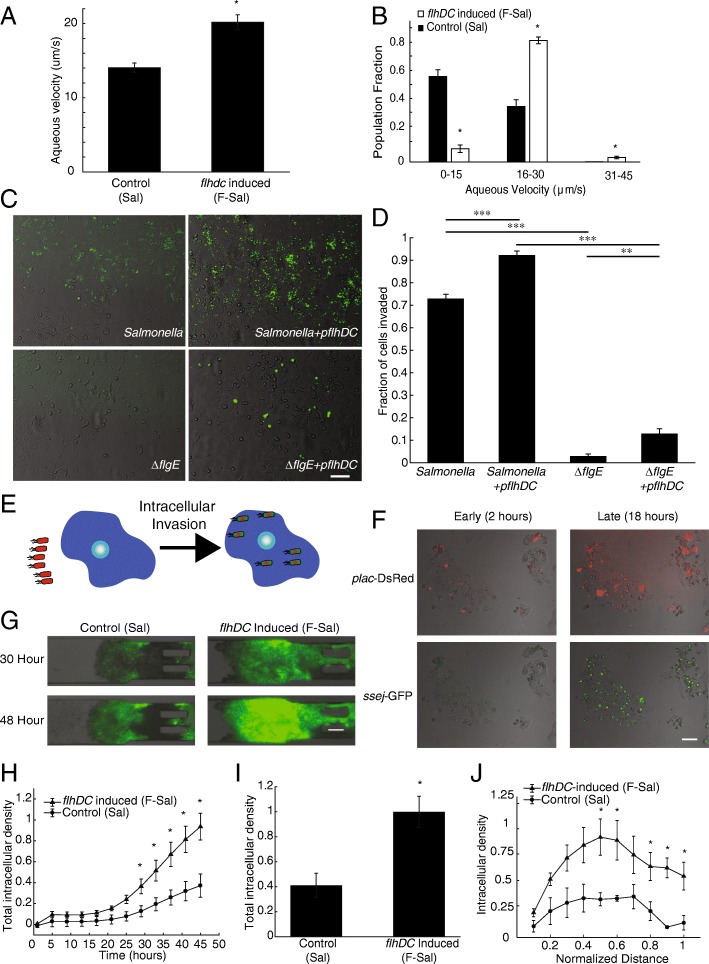
Induction *of flhDC* increases intracellular accumulation**. a)** After *flhDC* induction, *Salmonella* (F-Sal) were 33% more motile in aqueous solution than control *Salmonella* (Sal). **b)** In aqueous solution, the motile fraction of *Salmonella* (15-30 μm/s) increased while the non-motile fraction (0-15 um/s) decreased (*, *P* < 0.05). **c)** In monolayer culture, Salmonella (*green*) invaded into MCF7 cells. Salmonella with flagella (control and *pflhDC*) invaded cells more than non-motile (*ΔflgE* and *ΔflgE+pflhDC*) *Salmonella*. Some *ΔflgE+pflhDC Salmonella* invaded cells. All *Salmonella* constitutively expressed GFP. Scale bar is 100 μm. **d)** *Salmonella* overexpressing *flhDC* invaded cells 1.25 times more than control *Salmonella* (***, *P* < 0.001). *Salmonella* with intact flagella (control and *pflhDC*) invaded cells significantly more than non-flagellated (*ΔflgE* and *ΔflgE*+*pflhDC*) *Salmonella* (***, *P* < 0.001). Non-motile *ΔflgE*+*pflhDC Salmonella* invaded cells more than *ΔflgE Salmonella* (**, *P* <0.01). **e)** Four strains of *Salmonella* were transformed with *P*_*SSEJ*_*/GFP* and *P*_*lac*_*/DsRed* to identify extracellular (*red* only) and intracellular (*green* and *red*) bacteria. **f)** The *P*_*SSEJ*_ promoter is intracellularly activated. At an early time after invasion (2 hours), *Salmonella* only express *DsRed* (*top left)* and do not express *GFP* (*bottom left*). After 18 hours of incubation, intracellular *Salmonella* express both *GFP* (*bottom right*) and *DsRed* (*top right*). Scale bar is 100 μm. **g)** In tumor masses, many of the colonized *Salmonella* were intracellular. Scale bar is 100 μm. **h)** Overexpression of *flhDC* (F-Sal) increased the density of intracellular *Salmonella* in tumor masses 2.5 fold more than control *Salmonella* (Sal) at times greater than 29 hours after bacterial administration (*, *P* < 0.05). **i)** The average intracellular density of *flhDC* induced *Salmonella* was 2.5 fold greater than control *Salmonella* (*, *P* < 0.05). **j)** Induction of *flhDC* increased intracellular accumulation of F-Sal in medial (0.5 ≤ x ≤ 0.6) and distal (x ≥ 0.8) tumor tissue compared to controls (Sal; *, *P* < 0.05).

Upregulating *flhDC* in *Salmonella* increased intracellular accumulation in cells and tumor masses (Fig. 2). After induction with 0.2% arabinose, *Salmonella* motility increased by 25% (*P*<0.05, Fig. 2A). The non-motile fraction of bacteria (<15 μm/s) decreased seven-fold (*P*<0.01) and the motile fraction (>15 μm/s) increased two-fold (P<0.01, Fig. 2B).

In monolayer culture, *Salmonella* invaded into MCF7 cells and the extent of invasion was dependent on flagella (Fig. 2C). Overexpression of *flhDC* increased invasion 1.25 times compared to control *Salmonella* (*P* < 0.001, Fig. 2D). Invasion was highly dependent on functional flagella. Control *Salmonella* invaded cells 26-fold more than non-motile *ΔflgE Salmonella* (*P* < 0.001; Fig. 2D). Similarly, functional flagella had a large effect on cell invasion for Salmonella overexpressing *flhDC*; *pflhDC* Salmonella invaded 7.2 times more than *ΔflgE*+*pflhDC* Salmonella (*P* < 0.001). Flagella-independent invasion was increased 4.6 times by overexpression of *flhDC* (*P* < 0.01).

Four of the *Salmonella* strains (Sal, F-Sal, S-Sal and FS-Sal; Table 1) were transformed with *P*_*SSEJ*_*/GFP* (intracellular *GFP*) and *P*_*lac*_*/DsRed* (constitutive *DsRed*) to identify and differentiate total (*red* only) and intracellular (*red* and *green*) *Salmonella* (Fig. 2E). This genetic circuit is necessary in tumor cell masses, because constitutive fluorescence would not differentiate intracellular and extracellular bacteria. A gentamycin protection assay was used to show that *P*_*SSEJ*_ is a specific intracellular promoter. After applying control *Salmonella* (Sal) to a monolayer of cancer cells, all extracellular bacteria were removed with gentamycin. At early time points (2 h after gentamycin addition), *GFP* had yet to be translated (Fig. 2F, *lower left*) and all bacteria expressed *DsRed* (Fig. 2F, *upper left*). By 18 h, all intracellular bacteria (Fig. 2F, *upper right*) expressed both *DsRed* (Fig. 2F, *upper right)* and *GFP* (Fig. 2F, *lower right*), showing that the genetic circuits functioned as expected. In tumor-on-a-chip devices, overexpressing *flhDC* increased intracellular bacterial density (*green*, Fig. 2G). The high expression of GFP throughout the tumor masses (Fig. 2G) indicates that many of the *Salmonella* (both Sal and F-Sal) were intracellular (Additional file 2: Figure S2). Across all cell masses, the intracellular density of *flhDC*-induced F-Sal was significantly greater than control Sal from 29 to 45 h after administration (*P* < 0.05; Fig. 2H). Forty-eight hours after bacterial administration, the intracellular colonization of F-Sal was 2.5 fold more than Sal (*P*<0.05, Fig. 2I). In the middle of cell masses (0.5 < x < 0.6), induced F-Sal accumulated in cells 2.5 times more than control Sal (*P* < 0.05, Fig. 2J). Highly motile F-Sal also accumulated in distal tumor tissue (x ≥ 0.8) ten-fold more than Sal (*P*<0.05, Fig. 2J). These results demonstrate that *flhDC* induced *Salmonella* to accumulate in tumor cells.

### Induction of *flhDC* does not increase tumor colonization in the absence of intracellular accumulation

**Fig. 3:**
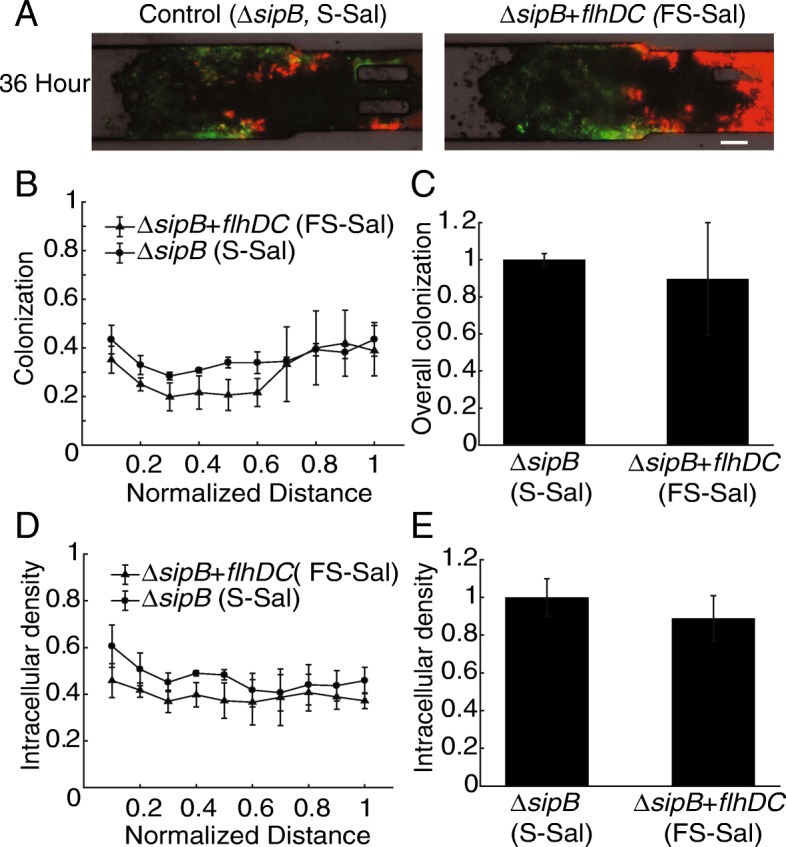
Induction of *flhDC* does not increase tumor colonization in the absence of T3SS1. **a)** In the absence of T3SS, extracellular (*red* only) and intracellular colonization (*green* and *red*) was minimal and uneven for *flhDC*-induced (FS-Sal) and control (S-Sal) *Salmonella*. Images were acquired 36 h after bacterial administration. Scale bar is 100 μm. **b-e)** When compared to control *ΔsipB Salmonella* (S-Sal), *flhDC*-induced *ΔsipB Salmonella* (FS-Sal) did not affect (**b**) the location of colonization, (**c**) the overall bacterial density, (**d**) the location of intracellular invasion, or (**e**) the overall extent of intracellular accumulation. Data (*n* = 3) were acquired 36 h after bacterial administration.

To investigate the effect of *flhDC* induction in the absence of T3SS-based invasion, ∆*sipB Salmonella* (S-Sal) were administered to a tumor-on-a-chip device (Fig. 3). No difference was seen in the colonization pattern of extracellular (*red*) or intracellular (*green*) *Salmonella* (Fig. 3A). Across multiple tumor cell masses (*n* = 3), no differences were observed in the location of *Salmonella* colonization after *flhDC* induction, based on *DsRed* expression (Fig. 3B), and there was no effect on total bacterial density (Fig. 3C). Similarly, *flhDC* induction did not affect the location of intracellular *Salmonella* based on *GFP* expression (Fig. 3D) or overall density of intracellular *Salmonella* (Fig. 3E). The lack of difference between FS-Sal and S-Sal indicates that *flhDC*-mediated intracellular accumulation requires a functional T3SS-I.

### Intracellular accumulation of *Salmonella* increases tumor colonization *in vitro*

**Fig. 4:**
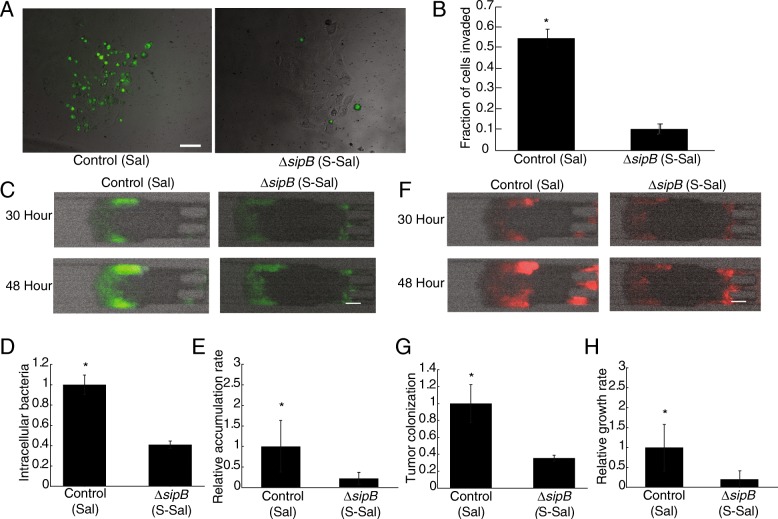
Tumor colonization of *Salmonella* depends on intracellular accumulation in tumor masses. **a**) Control *Salmonella* (Sal) intracellularly invaded MCF7 cells more than the minimally invasive *ΔsipB Salmonella* (S-Sal). Green fluorescence indicates induction of *GFP* expression by the *P*_*SSEJ*_ promoter, which is activated intracellularly. Scale bar is 100 μm. **b**) The *ΔsipB* mutant (S-Sal) intracellularly invaded tumor cells ten-fold less than control *Salmonella* in monolayer (*, P<0.05). **c**) The *sipB* knockout reduced the amount of intracellular *Salmonella* (*green*) in devices at 30 and 48 h after administration. Scale bar is 100 μm. **d, e**) Compared to control Sal, S-Sal (**d**) accumulated in tumor cells in devices 2.5 fold less (*, P<0.05, *n* = 6) and (**e**) had a four-fold slower rate of fluorescence increase (*, P<0.05). **f**) The *sipB* knockout also reduced the total density of colonized *Salmonella* (*red*) in devices at 30 and 48 h after administration. Scale bar is 100 μm. **g**, **h**) Compared to control (Sal), S-Sal (G) colonized tumors 2.5 fold less (*, *P*<0.05) and (**h**) grew in tumors four-fold slower (*, *P*<0.05).

Minimally invasive, *ΔsipB Salmonella* (S-Sal) colonized tumor tissue less than control *Salmonella* (Sal, Fig. 4). Both S-Sal and control Sal expressed *GFP* after intracellular invasion and constitutively expressed *DsRed* (Table 1). Without *sipB*, *Salmonella* invaded cancer cells considerably less than controls, as indicated by diminished *GFP* fluorescence (Fig. 4A). S-Sal invaded MCF-7 cells six-fold less than the Sal control (*P* < 0.05, Fig. 4B). When, S-Sal were administered to tumor-on-a-chip devices the amount of intracellular bacteria (*green*) was considerably less than for control Sal (Fig. 4C). The number of intracellular Sal increased from 30 to 48 hours as indicated by the increase in *GFP* intensity, but little increase was observed for S-Sal (Fig. 4C). Over multiple devices (*n* = 6), S-Sal accumulated within tumor masses 2.5 fold less than the Sal control (*P*<0.05, Fig. 4D) and the rate of *GFP* fluorescence increase of S-Sal was four fold less than Sal (*P*<0.05; Fig. 4E). Total tumor colonization was quantified through constitutive *DsRed* fluorescence. Thirty hours after administration, more control Sal bacteria were present in devices than S-Sal (Fig. 4F). The difference between Sal and S-Sal was due to the increase in intracellular invasion because knocking out *sipB* did not affect the growth rates of the strains (Additional file 3: Figure S3-A). Over multiple masses, S-Sal colonized tumor tissue four fold less (*P*<0.05, Fig. 4G) and grew four fold slower than the Sal control (*P*<0.05; Fig. 4H). Sal visibly grew between 30 and 48 hours after bacterial administration, while the S-Sal density remained relatively unchanged during the same time period (Fig. 4F). These results demonstrated that intracellular accumulation is an essential component of *Salmonella* tumor colonization *in vitro*.

### Intratumoral induction of *flhDC* improves colonization and intracellular accumulation of *Salmonella*

**Fig. 5:**
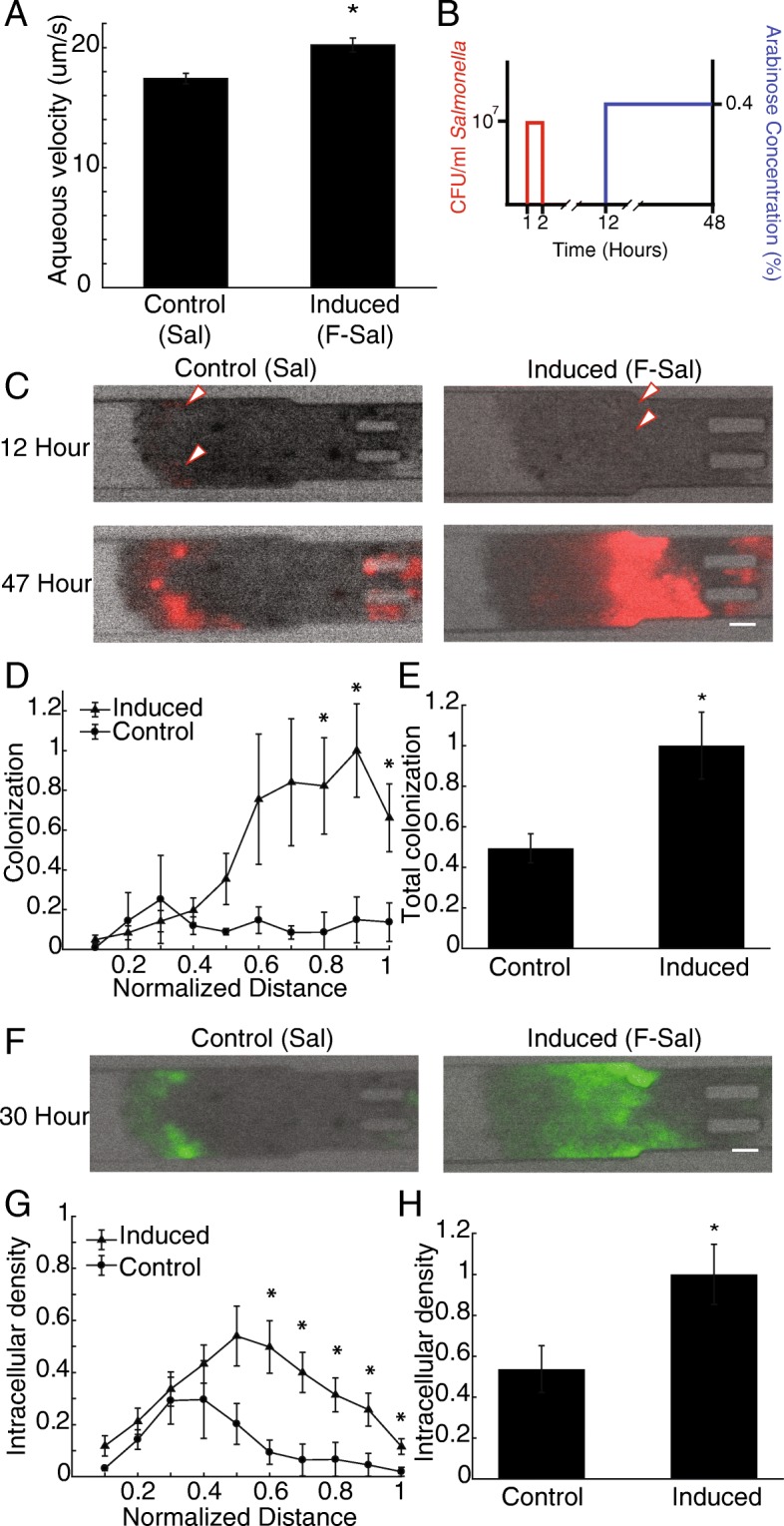
Intratumoral *flhDC* induction increases colonization, dispersion and intracellular accumulation of *Salmonella*. **a**) When *flhDC* was induced in *Salmonella,* aqueous motility increased by 18% compared to uninduced*Salmonella* containing the same pBAD-*flhDC* construct (*, *P*<0.05)*.* **b**) Graphical depiction of the dosing scheme. One hour after tumors were placed into devices, *Salmonella* was administered for 1 hour. Eleven hours after bacterial administration, media with 0.4% (w/v) arabinose was administered to the devices to induce bacterial *flhDC* expression. **c**) When F-Sal was administered to devices, bacteria colonies (red arrows) were first detected at 12 hours. At 47 h, colonies formed by F-Sal with intratumorally induced *flhDC* were larger than control *Salmonella* (Sal). Scale bar is 100 μm. **d**) Spatial distribution of intratumoral bacteria. Intratumoral induction of *flhDC* increased the level of distal bacterial colonization in tumor masses after 47 hours (*, *P*<0.05). **e**) Intratumoral induction of *flhDC* increased overall tumor colonization (*, *P*<0.05). **f**) Intratumorally induction of *flhDC* increased the number of intracellular *Salmonella* (*green*). Scale bar is 100 μm. **g**) Intratumoral *flhDC* expression increased intracellular accumulation in the distal region (0.6 < x < 1) of tumor masses (*, *P* < 0.05). **h**) Induction of *flhDC* increased intracellular accumulation within entire tumor masses after 36 hours (*, *P* < 0.05).

To determine if *flhDC* could be induced intratumorally, F-Sal was grown without arabinose and administered to tumor-on-a-chip devices. After induction with arabinose, F-Sal were 1.2 times faster in aqueous media compared to uninduced F-Sal (*P*<0.05; Fig. 5A). To test intratumoral induction, F-Sal were administered to devices for one hour in arabinose free medium (Fig. 5B). Twelve hours after administration, 0.4% arabinose added to the medium delivered in the flow channel to induce *flhDC* (Fig. 5B). Twelve hours was chosen as the time to induce, because this was the time when bacterial colonies could first be seen in the tumor cell masses (*red arrows*, Fig. 5C). At 47 h after administration, colonies grew in both uninduced and induced devices, but the induced colonies were visibly larger and located farther from the flow channel (Fig. 5C). Over multiple devices (*n* = 5 for uninduced and *n* = 6 for induced), intratumorally induced F-Sal colonized distal tumor tissue (0.8 ≤ x ≤ 1) five-fold more than the Sal control after 47 hours (*P*<0.05, Fig. 5D). The total amount of intratumorally induced F-Sal was two-fold greater than Sal (*P* <0.05, Fig. 5E).

Similar to overall density, induction increased the amount of intracellular F-Sal (Fig. 5F). Intracellular accumulation of intratumorally induced F-Sal was five-fold greater (*P*< 0.5) in intermediate tumor tissue (0.6 ≤ x ≤ 0.7) and two-fold greater (P< 0.5) in distal tumor tissue (0.8 ≤ x ≤ 1) compared to Sal (Fig. 5G). Total intracellular colonization of F-Sal was 1.8 fold greater than Sal after 30 hours (*P* <0.05, Fig. 5H). Intratumoral *flhDC* induction in *Salmonella* improved both distal colonization and intracellular accumulation when compared to *Salmonella* control, demonstrating that *flhDC* could be induced within tumors.

### Intracellular accumulation improves bacterial retention in tumors

A model of bacterial dispersion, growth and intracellular invasion was used to determine how modulating intracellular accumulation affected tumor colonization. The model includes balances on extracellular and intracellular bacteria (eq. 1-2). Extracellular bacteria (eq. 1) could accumulate, disperse, chemotax, invade cells, or be convectively transferred into the perfusion channel at the x = 0 boundary (eq. 3 *middle*). The number of intracellular bacteria increase because of either growth or cell invasion (eq. 2).

**Fig. 6:**
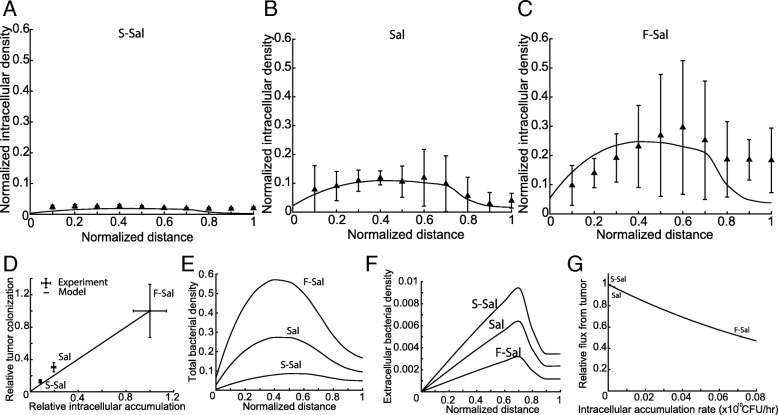
Intracellular accumulation increases retention of bacteria by preventing flux out of tumors. **a-c**) The mathematical model of intratumoral dispersion and invasion (eq 1-3) was fit to (**a**) *ΔsipB Salmonella* (S-Sal), (**b**) *Salmonella* (Sal)**,** and (**c**) *pflhDC*+*Salmonella* (F-Sal) to determine the intracellular accumulation rate of the three strains. The model was fit to all time points; images show the data and model fit at 31 h. **d**) The mathematical model fits experimental data and predicts that increasing intracellular accumulation would increase overall tumor colonization. **e**) The model predicts that increasing the rate of intracellular accumulation would increase overall tumor colonization, especially in intermediate tumor tissue (0.4 < x < 0.7). **f, g**) When the extracellular bacteria density is higher (compare S-Sal to F-Sal), there is a larger gradient at the front edge of the tumor (**f**), which causes more bacteria to leak out of tumors (**g**).

The model was used to calculate rates of intracellular accumulation and the bacterial dispersion coefficient in tumor masses. The model was fit to the spatiotemporal profiles of intracellular bacterial density for S-Sal, Sal and F-Sal (Fig. 6A-C). The dispersion coefficient (*D*) was calculated to be 23.5 μm^2^/s, by fitting to the Sal data set. The dispersion coefficient did not increase when the mathematical model was fit to the F-Sal dataset. The rate of intracellular accumulation for F-Sal was 4.47 times greater than Sal, and the accumulation rate of S-Sal was 2.39 times less than Sal (Table 2).

**Table 2:** Calculated Intracellular accumulation rates

Strain	Intracellular Accumulation Rate
S-Sal	1.8*x*10^7^ *CFU* ∗ *hr*^−1^
Sal	4.3*x*10^7^ *CFU* ∗ *hr*^−1^
F-Sal	19.2*x*10^7^ *CFU* ∗ *hr*^−1^

The model prediction of overall colonization as a function of the intracellular accumulation closely matched experimental data (Fig. 6D). When intracellular accumulation increased, overall tumor colonization increased. Theoretically extrapolating to bacteria that neither invade nor grow intracellularly suggests that they would not colonize tumors (Fig. 6D). Based on the model, the increase in bacterial density with higher rates of intracellular accumulation occurred primarily in intermediate regions of the cell masses (0.4 ≤ x ≤ 0.6; Fig. 6E). The calculated amounts of *extracellular* bacteria was greater for bacteria with lower rates of intracellular accumulation (i.e. S-Sal and Sal compared to F-Sal; Fig. 6F). Based on the model, this higher extracellular density (Fig. 6F) lead to greater leakage from the tumor and a lower overall density (Fig. 6G).

## Discussion

The results of this study demonstrate key mechanisms that control *Salmonella* colonization of tumors. Using *in vitro* tumors that can be monitored for bacterial infiltration and proliferation in real time, we demonstrated that overexpressing the master motility regulator, *flhDC*, increased tumor colonization (Fig. 1). As expected, induction of *flhDC* increased the motility of *Salmonella*, but it also increased the accumulation inside cancer cells (Fig. 2). In *Salmonella* with impaired invasiveness, *flhDC* induction did not affect colonization (Fig. 3) showing that *flhDC* enhances colonization by increasing the number of intracellular bacteria. Similarly, when *Salmonella* were modified to impair their invasiveness, tumor colonization was dramatically reduced (Fig. 4), showing that intracellular invasion and growth is important for *Salmonella* colonization of tumors, independent of *flhDC* overexpression. Integrating the spatial and temporal tumor penetration data into a mathematical model enabled calculation of the intracellular accumulation rate and showed that invasion promotes colonization by increasing bacterial retention in tumors (Fig. 6). These mechanisms could be used to improve therapeutic efficacy by enhancing bacterial tumor colonization. When *flhDC* was induced after initial penetration, intracellular accumulation and tumor colonization both increased (Fig. 5).

Overexpression of *flhDC* increased intracellular accumulation through a T3SS-dependent mechanism. When *flhDC* was upregulated in T3SS-deficient *Salmonella* (FS-Sal), neither intracellular accumulation nor colonization increased (Fig. 3B-E). Induction of *flhDC* increased T3SS-dependent intracellular accumulation primarily through flagella production and moderately through increased synthesis of T3SS components (Figs. 2 and 3). *Salmonella* that were incapable of producing flagella (*ΔflgE* and *ΔflgE+pflhDC*) accumulated significantly less than those able to assemble flagella (Fig. 2C, D). Overexpressing *flhDC* in *ΔflgE Salmonella* only marginally improved intracellular accumulation (Fig. 2D). The difference between these effects shows that the major contribution of *flhDC* was to produce flagella, which in turn improved accumulation. The increase in accumulation of non-motile *ΔflgE+pflhDC Salmonella*, however, shows that *flhDC* control of T3SS synthesis does play a role in controlling accumulation.

Two primary mechanisms could have increased intracellular accumulation after *flhDC* induction: cell invasion and intracellular growth. The T3SS1 system and functional flagella are important for both. The injection of T3SS1 effectors into mammalian cells is critical for cell invasion [29]. Similarly, T3SS1 plays an important role in the escape of *Salmonella* from intracellular vacuoles and hyper-replication in the cellular cytoplasm [45–47]. In addition to T3SS, invasion could have been mediated by alternate mechanisms, such as the EGFR-dependent Rck system. The contribution of alternate mechanisms was considerably less than the T3SS system (Fig. 4B). T3SS-deficient *Salmonella* (S-Sal) colonized tumor masses three-fold less than T3SS-competent control bacteria (Sal; Fig. 4G), although residual intracellular accumulation (Fig. 4D) and colonization (Fig. 4G) was observed.

The intracellular niche provides *Salmonella* with an environment to proliferate (Additional file 3: Figure S3B-C) and that is protected from convective clearance (Fig. 6G). In MCF7 cells in monolayers, *Salmonella* grew with a doubling time of 3.6 h (Additional file 3: Figure S3C), which is considerably faster than the doubling time within tumors in mice (16.8 h) [58]. Overexpressing *flhDC* increased bacterial density inside cells (Fig. 2D) and in distal tumor tissue (Fig. 1D). The fact that T3SS-deficient *Salmonella* accumulated far less in tumor masses than control *Salmonella* (Sal, Fig. 4F, G) suggests that intracellular and distal tumor tissue are protected from convective clearance (Fig. 6E, F).

The mathematical model of bacterial invasion and colonization shows how intracellular accumulation would improve bacterial retention (Fig. 6). Convection continuously clears bacteria from tumor tissue located near the perfusion channel (Fig. 6F). This mechanism is analogous to convective clearance of bacteria from tumors by the bloodstream. By invading tumor cells, fewer bacteria would reside extracellularly (Fig. 6F) and fewer would be cleared (Fig. 6G). As the rate of intracellular accumulation increases, more bacteria are retained within the tumor (Fig. 6D), a mechanism similar to the ‘binding’ of small-molecule drugs to cancer cells [59]. With small molecule drugs, it has been shown that drug/receptor binding improves retention within tumors once the drug clears from the blood [59]. By ‘binding’ to cancer cells, the model suggested that *Salmonella* with higher rates of intracellular accumulation are less prone to leaking out of tumors (Fig. 6G).

A distally located reservoir of extracellular bacteria could serve as a continuous source for intracellular invasion and colonization of tumors. Within *in vitro* tumor masses, there is a considerable amount of bacterial colonization in necrotic and quiescent tissue, which is located between necrotic and actively dividing tumor tissue [7]. Of the total population of colonized bacteria, the majority of extracellular bacteria were located in necrosis (Fig. 6F). Neither intracellular nor extracellular bacteria resided in tissue near the channel because of the high rate of convective clearance (Fig. 6E, F). Due to the high dispersion coefficient, extracellular bacteria would rapidly clear out of proximal tissue close to the perfusion channel. However, extracellular bacteria residing in necrosis could grow faster than the rate of dispersion (Fig. 6F) allowing for high regional accumulation and migration to viable tissue to invade cells.

**Fig. 7:**
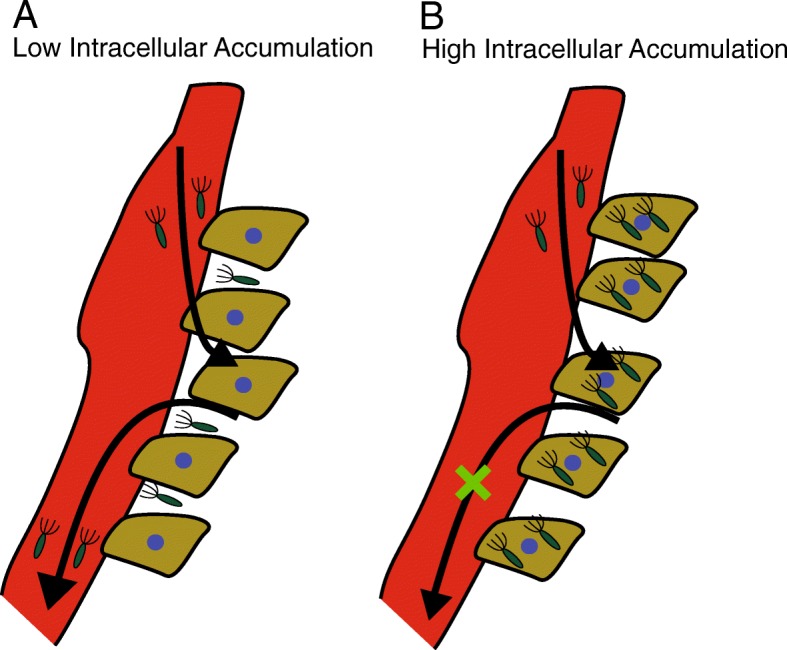
Graphical depiction of how intracellular accumulation could mechanistically improve tumor colonization. **a**) When *Salmonella* have a low intracellular accumulation rate, the rate of dispersion back into tumor vasculature is high, thus reducing bacterial tumor colonization due to a lack of “binding” to cancer cells. **b**) When *Salmonella* have a high intracellular accumulation rate, more bacteria would be retained in the tumor and not leak back into tumor microvasculature, thus increasing overall tumor colonization.

Controlling intracellular accumulation by inducing *flhDC* would increase tumor colonization. It would be beneficial to suppress flagellar expression outside of tumors. Flagella biosynthesis is an energetically costly process and can consume as much as 2% of bacterial energy [10, 60]. In addition *, Salmonella* flagellin is an immunogenic agonist that facilitates accelerated bacterial clearance [61]. Inducing *flhDC* selectively after initial penetration into tumors would improve fitness prior to administration, while promoting invasion and colonization within tumors (Fig. 7).

## Conclusion

This study demonstrates that overexpressing *flhDC* increases intracellular accumulation within tumor cell masses, which drives tumor colonization. Robust tumor colonization is necessary for *Salmonella* to be an effective drug delivery vehicle. Intracellular accumulation increased colonization by causing *Salmonella* to ‘bind’ to tumor cells. This binding prevented bacteria from being convectively cleared from tumor masses. Selectively inducing *flhDC* expression within tumor masses would promote fitness prior to administration and enhance colonization after initial penetration. We envision that therapeutic strains of *Salmonella* will utilize inducible *flhDC* to drive colonization in human tumors. After intravenous administration and a period of initial penetration, an inducer would be provided to activate the *flhDC* regulator. Intracellular invasion enables *Salmonella* to deliver a wide range therapies directly into the intracellular space of tumors. Measuring the mechanisms of intracellular bacterial accumulation and tumor colonization has identified a key regulator, *flhDC*, that could be used to amplify colonization and make *Salmonella* an effective anticancer therapeutic.

## Additional files

Additional file 1: **Figure S1.** The four plasmids used in this study. A) The control plasmid contains the PSSEJ/GFP and Plac/DsRed genetic circuits as well as chloramphenicol resistance and the ColE1 origin of replication. It was transformed into the Sal and S-Sal (ΔsipB) strains. B) The motility induction plasmid contains all of the components of the control plasmid (panel A) in addition to an arabinose inducible PBAD/flhDC genetic circuit. This plasmid was transformed into the F-Sal and FS-Sal strains. C) The constitutive GFP control plasmid contains the Plac/GFP genetic circuit, ampicillin resistance, and the ColE1 origin of replication. This plasmid was transformed into the control Salmonella and ΔflgE strains for measurement of cell invasion and intracellular growth. D) The motility induction, constitutive GFP plasmid contains all of the components of the constitutive GFP plasmid (panel C) in addition to an arabinose inducible PBAD/flhDC genetic circuit. This plasmid was transformed into the Salmonella+pflhDC and ΔflgE+pflhDC strains for measurement of cell invasion and intracellular growth. (PDF 955 kb)

Additional file 2: **Figure S2.** Merged fluorescent images of intratumoral Salmonella. Merged fluorescent images of intratumoral Salmonella. DsRed indicates the presence of all bacteria while GFP indicates the presence of intracellular bacteria. DsRed images have been enhanced to visualize all intratumoral bacteria. (PDF 3750 kb)

Additional file 3: **Figure S1.** Growth Rates of Salmonella. A) Growth rate of Salmonella in liquid media (LB). All three strains grew at about the same rate (Sal, 1.313 hr-1; F-Sal, 1.273 hr-1; S-Sal; 1.26 hr-1), although F-Sal grew at a significantly slower rate than Sal (*, *P* < 0.05). There was no difference in the growth rates of ΔsipB (S-Sal) and control (Sal). B) Constitutive GFP fluorescence of intracellular Salmonella within MCF7 cells. The increase in intensity from one to five hours indicates the increase in the number of bacteria. Scale bar is 10 μm. C) Intracellular bacteria grew exponentially at a rate of 0.19 hr^-1^. (PDF 950 kb)

## List of abbreviations

*flhDC*: *Salmonella* master motility regulator
T3SS1: Type three secretion system-1
T3SS2: Type three secretion system-2
fT3SS: Flagellar type three secretion system
*sipB*: Type three secretion system cap protein
*GFP*: Green fluorescent protein
*DsRed*: A red fluorescent protein
SSEJ-*GFP*: Intracellular GFP expression genetic circuit
Lac-*DsRed*: Constitutive red fluorescent protein expression
F-Sal: *Salmonella* transformed with *SSEJ-GFP* and *Lac-DsRed*
Sal: *Salmonella* transformed with *SSEJ-GFP* and *Lac-DsRed*
S-Sal: *ΔsipB Salmonella* transformed with *Lac-DsRed*
FS-Sal: *ΔsipB Salmonella* transformed with *SSEJ-GFP*
*Lac*: *DsRed* and *PBAD-flhDC*
DMEM: Dulbecco’s minimal eagle medium
FBS: Fetal bovine serum
PMMA: Poly-(methyl)-methacrylate
PDMS: Poly-(dimethyl)-siloxane
HEPES: (4-(2-hydroxyethyl)-1-piperazineethanesulfonic acid)
CFU: Colony forming unit
LB: Luria Bertani broth

## References

[1] Kocijancic D, Felgner S, Schauer T, Frahm M, Heise U, Zimmermann K, Erhardt M, Weiss S (2017). **L**ocal application of bacteria improves safety of Salmonella-mediated tumor therapy and retains advantages of systemic infection. Oncotarget.

[2] Forbes NS, Munn LL, Fukumura D, Jain RK (2003). Sparse initial entrapment of systemically injected Salmonella typhimurium leads to heterogeneous accumulation within tumors. Cancer Res.

[3] Forbes NS (2010). Engineering the perfect (bacterial) cancer therapy. Nature Reviews Cancer.

[4] Toso JF, Gill VJ, Hwu P, Marincola FM, Restifo NP, Schwartzentruber DJ, Sherry RM, Topalian SL, Yang JC, Stock F (2002). Phase I study of the intravenous administration of attenuated Salmonella typhimurium to patients with metastatic melanoma. J Clin Oncol.

[5] Toley BJ, Forbes NS (2012). Motility is critical for effective distribution and accumulation of bacteria in tumor tissue. Integr Biol (Camb).

[6] Thornlow DN, Brackett EL, Gigas JM, Van Dessel N, Forbes NS (2015). Persistent enhancement of bacterial motility increases tumor penetration. Biotechnol Bioeng.

[7] Zhang M, Forbes NS (2015). Trg-deficient Salmonella colonize quiescent tumor regions by exclusively penetrating or proliferating. J Control Release.

[8] Silva-Valenzuela CA, Desai PT, Molina-Quiroz RC, Pezoa D, Zhang Y, Porwollik S, Zhao M, Hoffman RM, Contreras I, Santiviago CA, McClelland M (2016). Solid tumors provide niche-specific conditions that lead to preferential growth of Salmonella. Oncotarget.

[9] Kasinskas RW, Forbes NS (2007). Salmonella typhimurium lacking ribose chemoreceptors localize in tumor quiescence and induce apoptosis. Cancer Res.

[10] Gauger EJ, Leatham MP, Mercado-Lubo R, Laux DC, Conway T, Cohen PS (2007). Role of motility and the flhDC Operon in Escherichia coli MG1655 colonization of the mouse intestine. Infect Immun.

[11] Wang X, Wood TK (2011). IS5 inserts upstream of the master motility operon flhDC in a quasi-Lamarckian way. ISME J.

[12] Macnab RM (1992). Genetics and biogenesis of bacterial flagella. Annu Rev Genet.

[13] Liu X, Matsumura P (1994). The FlhD/FlhC complex, a transcriptional activator of the Escherichia coli flagellar class II operons. J Bacteriol.

[14] Clarke MB, Sperandio V (2005). Transcriptional regulation of flhDC by QseBC and sigma (FliA) in enterohaemorrhagic Escherichia coli. Mol Microbiol.

[15] Singer HM, Kuhne C, Deditius JA, Hughes KT, Erhardt M (2014). The Salmonella Spi1 virulence regulatory protein HilD directly activates transcription of the flagellar master operon flhDC. J Bacteriol.

[16] Soutourina O, Kolb A, Krin E, Laurent-Winter C, Rimsky S, Danchin A, Bertin P (1999). Multiple control of flagellum biosynthesis in Escherichia coli: role of H-NS protein and the cyclic AMP-catabolite activator protein complex in transcription of the flhDC master operon. J Bacteriol.

[17] Sperandio V, Torres AG, Kaper JB (2002). Quorum sensing Escherichia coli regulators B and C (QseBC): a novel two-component regulatory system involved in the regulation of flagella and motility by quorum sensing in E. coli. Mol Microbiol.

[18] Wei BL, Brun-Zinkernagel AM, Simecka JW, Pruss BM, Babitzke P, Romeo T (2001). Positive regulation of motility and flhDC expression by the RNA-binding protein CsrA of Escherichia coli. Mol Microbiol.

[19] Yakhnin AV, Baker CS, Vakulskas CA, Yakhnin H, Berezin I, Romeo T, Babitzke P (2013). CsrA activates flhDC expression by protecting flhDC mRNA from RNase E-mediated cleavage. Mol Microbiol.

[20] Wada T, Hatamoto Y, Kutsukake K (2012). Functional and expressional analyses of the anti-FlhD4C2 factor gene ydiV in Escherichia coli. Microbiology.

[21] Schlumberger MC, Hardt WD (2006). Salmonella type III secretion effectors: pulling the host cell's strings. Curr Opin Microbiol.

[22] Wiedemann A, Mijouin L, Ayoub MA, Barilleau E, Canepa S, Teixeira-Gomes AP, Le Vern Y, Rosselin M, Reiter E, Velge P (2016). Identification of the epidermal growth factor receptor as the receptor for Salmonella Rck-dependent invasion. Faseb Journal.

[23] Hapfelmeier S, Stecher B, Barthel M, Kremer M, Muller AJ, Heikenwalder M, Stallmach T, Hensel M, Pfeffer K, Akira S, Hardt WD (2005). The Salmonella pathogenicity island (SPI)-2 and SPI-1 type III secretion systems allow Salmonella serovar typhimurium to trigger colitis via MyD88-dependent and MyD88-independent mechanisms. J Immunol.

[24] Pawelek JM, Sodi S, Chakraborty AK, Platt JT, Miller S, Holden DW, Hensel M, Low KB (2002). Salmonella pathogenicity island-2 and anticancer activity in mice. Cancer Gene Ther.

[25] Arrach N, Cheng P, Zhao M, Santiviago CA, Hoffman RM, McClelland M (2010). High-throughput screening for salmonella avirulent mutants that retain targeting of solid tumors. Cancer Res.

[26] Koskiniemi S, Gibbons HS, Sandegren L, Anwar N, Ouellette G, Broomall S, Karavis M, McGregor P, Liem A, Fochler E (2013). Pathoadaptive mutations in Salmonella enterica isolated after serial passage in mice. PLoS One.

[27] Dumoux M, Nans A, Saibil HR, Hayward RD (2015). Making connections: snapshots of chlamydial type III secretion systems in contact with host membranes. Curr Opin Microbiol.

[28] Lucas RL, Lee CA (2001). Roles of hilC and hilD in regulation of hilA expression in Salmonella enterica serovar Typhimurium. J Bacteriol.

[29] Myeni SK, Wang L, Zhou D (2013). SipB-SipC complex is essential for translocon formation. PLoS One.

[30] Van Engelenburg SB, Palmer AE (2010). Imaging type-III secretion reveals dynamics and spatial segregation of Salmonella effectors. Nat Methods.

[31] Galan JE, Wolf-Watz H (2006). Protein delivery into eukaryotic cells by type III secretion machines. Nature.

[32] Hayward RD, McGhie EJ, Koronakis V (2000). Membrane fusion activity of purified SipB, a Salmonella surface protein essential for mammalian cell invasion. Mol Microbiol.

[33] Knodler LA, Vallance BA, Hensel M, Jackel D, Finlay BB, Steele-Mortimer O (2003). Salmonella type III effectors PipB and PipB2 are targeted to detergent-resistant microdomains on internal host cell membranes. Mol Microbiol.

[34] Uchiya K, Barbieri MA, Funato K, Shah AH, Stahl PD, Groisman EA (1999). A Salmonella virulence protein that inhibits cellular trafficking. EMBO J.

[35] Meresse S, Unsworth KE, Habermann A, Griffiths G, Fang F, Martinez-Lorenzo MJ, Waterman SR, Gorvel JP, Holden DW (2001). Remodelling of the actin cytoskeleton is essential for replication of intravacuolar Salmonella. Cell Microbiol.

[36] Birmingham CL, Jiang X, Ohlson MB, Miller SI, Brumell JH (2005). Salmonella-induced filament formation is a dynamic phenotype induced by rapidly replicating Salmonella enterica serovar typhimurium in epithelial cells. Infect Immun.

[37] Guignot J, Caron E, Beuzon C, Bucci C, Kagan J, Roy C, Holden DW (2004). Microtubule motors control membrane dynamics of Salmonella-containing vacuoles. J Cell Sci.

[38] Ribet D, Cossart P (2015). How bacterial pathogens colonize their hosts and invade deeper tissues. Microbes Infect.

[39] Chubiz JE, Golubeva YA, Lin D, Miller LD, Slauch JM (2010). FliZ regulates expression of the Salmonella pathogenicity island 1 invasion locus by controlling HilD protein activity in Salmonella enterica serovar typhimurium. J Bacteriol.

[40] Mouslim C, Hughes KT (2014). The effect of cell growth phase on the regulatory cross-talk between flagellar and Spi1 virulence gene expression. PLoS Pathog.

[41] Elhadad D, Desai P, Rahav G, McClelland M, Gal-Mor O (2015). Flagellin Is Required for Host Cell Invasion and Normal Salmonella Pathogenicity Island 1 Expression by Salmonella enterica Serovar Paratyphi A. Infect Immun.

[42] Misselwitz B, Barrett N, Kreibich S, Vonaesch P, Andritschke D, Rout S, Weidner K, Sormaz M, Songhet P, Horvath P (2012). Near surface swimming of Salmonella Typhimurium explains target-site selection and cooperative invasion. PLoS Pathog.

[43] Abby SS, Rocha EP (2012). The non-flagellar type III secretion system evolved from the bacterial flagellum and diversified into host-cell adapted systems. PLoS Genet.

[44] Lee SH, Galan JE (2004). Salmonella type III secretion-associated chaperones confer secretion-pathway specificity. Mol Microbiol.

[45] Knodler LA, Vallance BA, Celli J, Winfree S, Hansen B, Montero M, Steele-Mortimer O (2010). Dissemination of invasive Salmonella via bacterial-induced extrusion of mucosal epithelia. Proc Natl Acad Sci U S A.

[46] Wrande M, Andrews-Polymenis H, Twedt DJ, Steele-Mortimer O, Porwollik S, McClelland M, Knodler LA (2016). Genetic Determinants of Salmonella enterica Serovar Typhimurium Proliferation in the Cytosol of Epithelial Cells. Infect Immun.

[47] Knodler LA, Nair V, Steele-Mortimer O (2014). Quantitative assessment of cytosolic Salmonella in epithelial cells. PLoS One.

[48] van Wijk SJL, Fricke F, Herhaus L, Gupta J, Hotte K, Pampaloni F, Grumati P, Kaulich M, Sou YS, Komatsu M (2017). Linear ubiquitination of cytosolic Salmonella Typhimurium activates NF-kappaB and restricts bacterial proliferation. Nat Microbiol.

[49] Fiskin E, Bionda T, Dikic I, Behrends C (2016). Global Analysis of Host and Bacterial Ubiquitinome in Response to Salmonella Typhimurium Infection. Mol Cell.

[50] Wild P, Farhan H, McEwan DG, Wagner S, Rogov VV, Brady NR, Richter B, Korac J, Waidmann O, Choudhary C (2011). Phosphorylation of the autophagy receptor optineurin restricts Salmonella growth. Science.

[51] Thurston TL, Ryzhakov G, Bloor S, von Muhlinen N, Randow F (2009). The TBK1 adaptor and autophagy receptor NDP52 restricts the proliferation of ubiquitin-coated bacteria. Nat Immunol.

[52] Cemma M, Kim PK, Brumell JH (2011). The ubiquitin-binding adaptor proteins p62/SQSTM1 and NDP52 are recruited independently to bacteria-associated microdomains to target Salmonella to the autophagy pathway. Autophagy.

[53] Datsenko KA, Wanner BL (2000). One-step inactivation of chromosomal genes in Escherichia coli K-12 using PCR products. Proc Natl Acad Sci U S A.

[54] Deditius JA, Felgner S, Sporing I, Kuhne C, Frahm M, Rohde M, Weiss S, Erhardt M (2015). Characterization of Novel Factors Involved in Swimming and Swarming Motility in Salmonella enterica Serovar Typhimurium. PLoS One.

[55] Walsh CL, Babin BM, Kasinskas RW, Foster JA, McGarry MJ, Forbes NS (2009). A multipurpose microfluidic device designed to mimic microenvironment gradients and develop targeted cancer therapeutics. Lab Chip.

[56] Mohan R, Schudel BR, Desai AV, Yearsley JD, Apblett CA, Kenis PJA (2011). Design considerations for elastomeric normally closed microfluidic valves. Sensors and Actuators B-Chemical.

[57] Kasinskas RW, Forbes NS (2006). Salmonella typhimurium specifically chemotax and proliferate in heterogeneous tumor tissue in vitro. Biotechnol Bioeng.

[58] Ganai S, Arenas RB, Sauer JP, Bentley B, Forbes NS (2011). In tumors Salmonella migrate away from vasculature toward the transition zone and induce apoptosis. Cancer Gene Ther.

[59] Toley BJ, Tropeano Lovatt ZG, Harrington JL, Forbes NS (2013). Microfluidic technique to measure intratumoral transport and calculate drug efficacy shows that binding is essential for doxorubicin and release hampers Doxil. Integr Biol (Camb).

[60] Soutourina OA, Bertin PN (2003). Regulation cascade of flagellar expression in Gram-negative bacteria. FEMS Microbiol Rev.

[61] Yang X, Thornburg T, Suo Z, Jun S, Robison A, Li J, Lim T, Cao L, Hoyt T, Avci R, Pascual DW (2012). Flagella overexpression attenuates Salmonella pathogenesis. PLoS One.

