# Supplementary material for: The motility regulator flhDC drives intracellular accumulation and tumor colonization of Salmonella
Source: J Immunother Cancer. 2019 Feb 12;7:44. doi: 10.1186/s40425-018-0490-z (PMC6373116; doi:10.1186/s40425-018-0490-z)
Supplement: Supplementary file 1 — Figure S1. The four plasmids used in this study. A) The control plasmid contains the PSSEJ/GFP and Plac/DsRed genetic circuits as well as chloramphenicol resistance and the ColE1 origin of replication. It was transformed into the Sal and S-Sal (ΔsipB) strains. B) The motility induction plasmid contains all of the components of the control plasmid (panel A) in addition to an arabinose inducible PBAD/flhDC genetic circuit. This plasmid was transformed into the F-Sal and FS-Sal strains. C) The constitutive GFP control plasmid contains the Plac/GFP genetic circuit, ampicillin resistance, and the ColE1 origin of replication. This plasmid was transformed into the control Salmonella and ΔflgE strains for measurement of cell invasion and intracellular growth. D) The motility induction, constitutive GFP plasmid contains all of the components of the constitutive GFP plasmid (panel C) in addition to an arabinose inducible PBAD/flhDC genetic circuit. This plasmid was transformed into the Salmonella+pflhDC and ΔflgE+pflhDC strains for measurement of cell invasion and intracellular growth. (PDF 955 kb) [file 40425_2018_490_MOESM1_ESM.pdf]

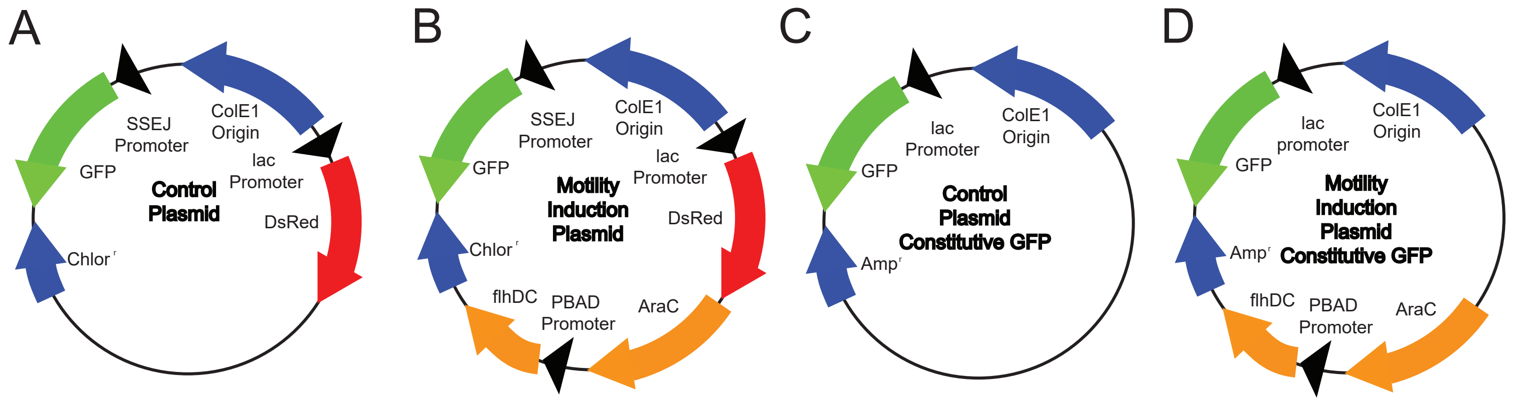

Figure S1. The four plasmids used in this study. A) The control plasmid contains the P_{SSEJ}*/GFP* and P_{lac}*/DsRed* genetic circuits as well as chloramphenicol resistance and the ColE1 origin of replication. It was transformed into the Sal and S-Sal (Δ*sipB*) strains. **B)** The motility induction plasmid contains all of the components of the control plasmid (panel *A*) in addition to an arabinose inducible P_{BAD}*/flhDC* genetic circuit. This plasmid was transformed into the F-Sal and FS-Sal strains. **C)** The constitutive GFP control plasmid contains the P_{lac}*/GFP* genetic circuit, ampicillin resistance, and the ColE1 origin of replication. This plasmid was transformed into the control *Salmonella* and *ΔflgE* strains for measurement of cell invasion and intracellular growth. **D)** The motility induction, constitutive GFP plasmid contains all of the components of the constitutive GFP plasmid (panel *C*) in addition to an arabinose inducible P_{BAD}*/flhDC* genetic circuit. This plasmid was transformed into the *Salmonella+pflhDC* and *ΔflgE+pflhDC* strains for measurement of cell invasion and intracellular growth.
