# Supplementary material for: The motility regulator flhDC drives intracellular accumulation and tumor colonization of Salmonella
Source: J Immunother Cancer. 2019 Feb 12;7:44. doi: 10.1186/s40425-018-0490-z (PMC6373116; doi:10.1186/s40425-018-0490-z)
Supplement: Supplementary file 2 — Figure S2. Merged fluorescent images of intratumoral Salmonella. Merged fluorescent images of intratumoral Salmonella. DsRed indicates the presence of all bacteria while GFP indicates the presence of intracellular bacteria. DsRed images have been enhanced to visualize all intratumoral bacteria. (PDF 3750 kb) [file 40425_2018_490_MOESM2_ESM.pdf]

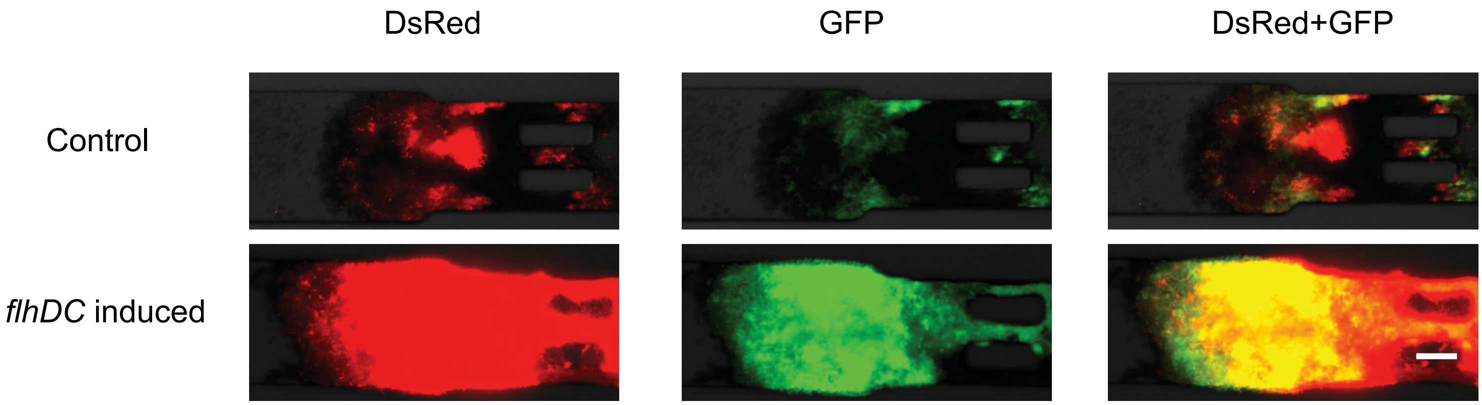

Figure S2. **Merged fluorescent images of intratumoral *Salmonella*.** Merged fluorescent images of intratumoral *Salmonella*. DsRed indicates the presence of all bacteria while GFP indicates the presence of intracellular bacteria. DsRed images have been enhanced to visualize all intratumoral bacteria.
