# Supplementary material for: The motility regulator flhDC drives intracellular accumulation and tumor colonization of Salmonella
Source: J Immunother Cancer. 2019 Feb 12;7:44. doi: 10.1186/s40425-018-0490-z (PMC6373116; doi:10.1186/s40425-018-0490-z)
Supplement: Supplementary file 3 — Figure S1. Growth Rates of Salmonella. A) Growth rate of Salmonella in liquid media (LB). All three strains grew at about the same rate (Sal, 1.313 hr-1; F-Sal, 1.273 hr-1; S-Sal; 1.26 hr-1), although F-Sal grew at a significantly slower rate than Sal (*, P < 0.05). There was no difference in the growth rates of ΔsipB (S-Sal) and control (Sal). B) Constitutive GFP fluorescence of intracellular Salmonella within MCF7 cells. The increase in intensity from one to five hours indicates the increase in the number of bacteria. Scale bar is 10 μm. C) Intracellular bacteria grew exponentially at a rate of 0.19 hr-1. (PDF 950 kb) [file 40425_2018_490_MOESM3_ESM.pdf]

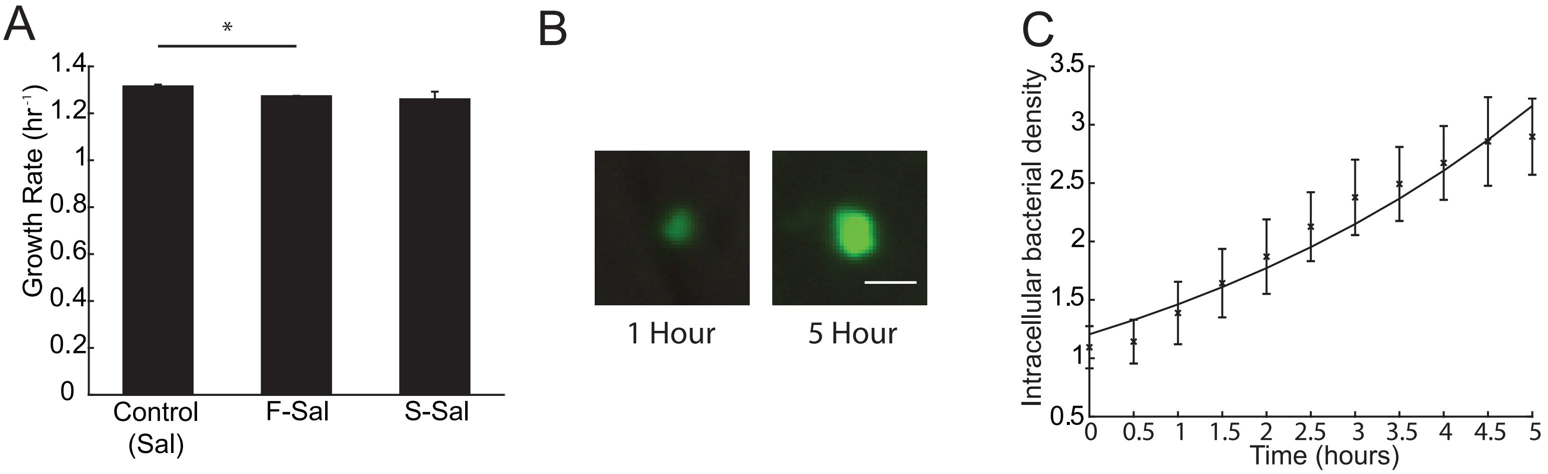

Figure S3. Growth Rates of *Salmonella*. A) Growth rate of *Salmonella* in liquid media (LB). All three strains grew at about the same rate (Sal, 1.313 hr$_{-1}$; F-Sal, 1.273 hr$_{-1}$; S-Sal; 1.26 hr$_{-1}$), although F-Sal grew at a slower significantly slower rate than Sal (*, $P < 0.05$). There was no difference in the growth rates of *ΔsipB* (S-Sal) and control (Sal). **B)** Intracellular bacteria grew exponentially at a rate of 0.19 hr^{-1}. **(C)** The mathematical model closely fits experimental data and predicts that increasing intracellular accumulation would increase overall tumor colonization.
